# Probing Glass Formation in Perylene Derivatives via Atomic-Scale Simulations and Bayesian Regression

**DOI:** 10.1021/acs.jpcb.5c00837

**Published:** 2025-06-23

**Authors:** Eric Lindgren, Jan Swenson, Christian Müller, Paul Erhart

**Affiliations:** † Department of Physics, 11248 Chalmers University of Technology, Gothenburg SE-41296, Sweden; ‡ Department of Chemistry and Chemical Engineering, 11248 Chalmers University of Technology, Gothenburg SE-41296, Sweden

## Abstract

While the structural dynamics of chromophores are of interest for a range of applications, it is experimentally very challenging to resolve the underlying microscopic mechanisms. At the same time, glassy dynamics are also challenging for atomistic simulations due to the underlying dramatic slowdown over many orders of magnitude. Here, we address this issue by combining atomic scale simulations with autocorrelation function analysis and Bayesian regression, and apply this approach to a set of perylene derivatives as prototypical chromophores. The predicted glass transition temperatures and kinetic fragilities are in semiquantitative agreement with experimental data. We suggest that the remaining error could be caused by an overestimation of the intermolecular cohesion by the force field used in this work. By analyzing the underlying dynamics via the normal vector autocorrelation function, we are able to connect the β and α-relaxation processes in these materials to caged (or librational) dynamics and cooperative rotations of the molecules, respectively. The workflow presented in this work serves as a stepping stone toward understanding glassy dynamics in many-component mixtures of perylene derivatives and is readily extendable to other systems of chromophores.

## Graphical abstract

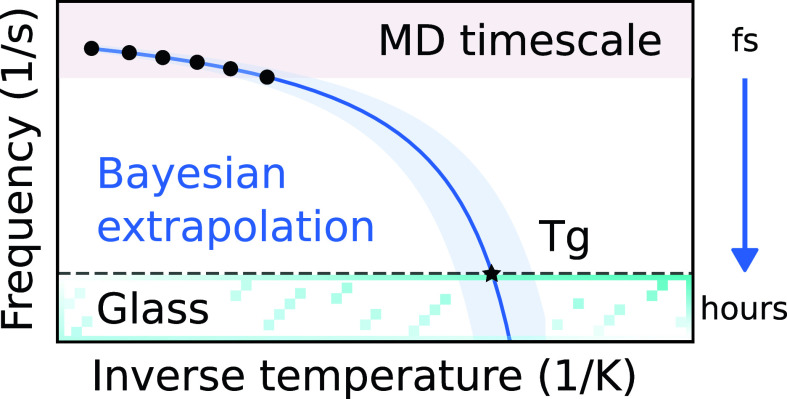

## Introduction

Chromophores are an important class of materials with a range of potential and realized applications in the area of energy conversion thanks to their exceptional optical properties. Chromophores have been studied, e.g., as active materials in solar cells,^1–5^ organic light-emitting diodes,^6,7^ and photoswitchable and solar thermal storage systems.^8–10^ The properties of these materials are sensitive to both the structural arrangements of the molecules and their dynamic behavior. The dynamics as manifested in macroscopic properties such as viscosity and diffusivity, are also important for solution processing, which is currently the most common approach for large-scale manufacturing of devices based on these materials. Controlling viscosity and diffusivity is often achieved through glass formation,^11^ which can occur upon rapid cooling, bypassing crystallization and resulting in a glassy state that lacks long-range order. The glass transition is characterized by a dramatic slow down in the materials dynamics over a narrow temperature range that is commonly probed via the temperature dependence of, e.g., the viscosity (via rheometry), the density (via dilatometry) or the heat exchanged with the environment (via calorimetry).

For practical use, it is crucial to achieve glass formation controllably at modest cooling rates. In this context, using mixtures of perylene derivatives, it has been shown that increasingly stronger glass formers can be systematically obtained by increasing the number of components. This principle works even though the underlying molecules are weak (“fragile”) glass formers in single-component systems.^12^ Moreover, it has been found that such many-component mixtures have further benefits, including significantly improved thermal stability.^13^ While many-components mixtures thus have very high potential for materials design, the much larger design space also renders understanding the underlying dynamical processes much more challenging. Here, as a first step toward a systematic understanding of these materials, we investigate glass formation in single-component liquids of perylene derivatives (Figure 1a) using molecular dynamics (MD) simulations in combination with Bayesian regression.

**Figure 1:**
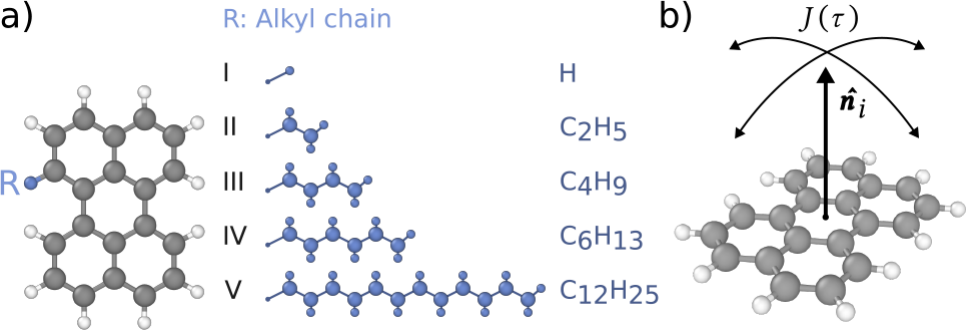
(a) Perylene derivatives studied in this work. (b) Schematic representation of the normal vector autocorrelation function *J*(τ), see eq 2.

While the microscopic dynamics of glass-forming systems can be explored via MD simulations,^14,15^ it is usually challenging to directly access the temperature range in which the glass transition occurs due the time-scale limitations of this technique, although there are cases in which it is possible.^16^ Here, to extend the temperature range, we combine MD simulations with Bayesian regression, which allows us to predict glass transition temperatures as well as the propensity for glass formation (expressed via the kinetic fragility). To this end, we observe the temperature dependence of the dynamics via the diffusivity, which is anticorrelated with the viscosity but computationally easier to converge than the latter. Our results for the glass transition temperatures and the kinetic fragility are in semiquantitative agreement with experimental data, supporting the viability of the simulation approach. To gain insight into the microscopic processes we analyze the time-autocorrelation function (ACF) of the molecular orientation, which reveals three distinct dynamic regimes corresponding to intramolecular motion as well as β and α-relaxation processes. Our work thereby establishes the viability of this simulation approach and lays the groundwork for future studies of the evolution of the dynamics in many-component mixtures.

## Methods

We considered five perylene derivatives (Figure 1a), which differ with respect to the length *n* of the pendant alkyl chain C_*n*_H_2*n*+1_ attached to one of the bay positions. Monomer **I** corresponds to regular perylene with no alkyl chain, whereas monomers **II–V** have alkyl chains containing two (*n* = 2), four (*n* = 4), six (*n* = 6), and 12 (*n* = 12) carbon atoms, respectively.

### Diffusivity and Autocorrelation Functions

To characterize the dynamics of the perylene derivatives, we first consider the molecular diffusivity *D*, which can be obtained from the MSD ⟨Δ*r* ^2^⟩ of the molecular centroid positions^17^

$$\langle \Delta r^{2}\rangle =6D\tau$$ 1

The diffusivity was computed using production runs with a duration of up to 10 ns.

To obtain more detailed insight into the underlying microscopic properties, we also analyzed the ACF of the normal vectors indicating the orientation of each individual molecule (Figure 1b) given by

$$J\left(\tau \right)={\left\langle {\mathrm{n̂}}_{i}\left(t\right)\cdot {\mathrm{n̂}}_{i}\left(t+\tau \right)\right\rangle }_{it}$$ 2

Here, ***n̂***_*i*_(*t*) is the normal vector of molecule *i* (Section S1). The ensemble average applies over all times *t* and each molecule *i* in the system. Equation 2 can be efficiently evaluated using the Wiener–Kinchin theorem.

One may also extract the standard error as an uncertainty estimate for *J*(τ) from the ACF for each molecule *J*_*i*_(τ) before computing the ensemble average in eq 2 according to

$$\sigma_{J}(\tau )=\sqrt{\mathrm{Var}(\{J_{i}{\}}_{i=1}^{N})/N}$$ 3

where *N* is the number of molecules in the system.

Since the ACFs spans multiple orders of magnitude in time, production runs of different length were conducted. To sample short and long-time scales, simulations with a length of respectively 100 ps and 10 ns were carried out with snapshots being written every 1 and 100 fs, respectively (Section S1). The normal vector ACFs were calculated for both production runs and subsequently spliced together at a time lag of 1 ps.

### Computational Details

MD simulations were performed using the gromacs package^18^ (version 2021.3) with the OPLS all-atom force field.^19^ Topology and structure files where generated using the LigParGen server,^20–22^ starting from structures from the automated topology builder and repository.^23–25^ The per-atom charges obtained from the LigParGen server where slightly modified in order to obtain neutral molecules. A time step of 1 fs was used for all simulations, in combination with constraining the hydrogen atoms using the linear constraint solver algorithm.^26^ The simulation cell contained between 500 and 2000 molecules depending on the length of the alkyl chain of the perylene derivative, and simulations were performed at temperatures in the range 400–800 K.

Each system was equilibrated at the target temperature prior to production using the following protocol. First, the system energy was minimized using a steepest descent optimizer, after which a simulation of 1 ns was performed in the *NVT* ensemble. This was followed by a 1 ns run in the *NPT* ensemble at a pressure of 2 kbar using a Berendsen barostat^27^ to avoid cavitation. The high-pressure *NPT* simulation was followed by a 10 ns *NPT* simulation at 1 bar. Finally, production runs were carried out in the *NPT* ensemble using the stochastic pressure-rescaling barostat and a stochastic velocity-rescaling thermostat^28^ to obtain the diffusivity as well as the short and long-time normal vector ACF (Section S1). The production runs were 100 ps and 10 ns long, and trajectory files were written every 1 and 100 fs, respectively.

The trajectories resulting from the simulations were then parsed using the mdtraj package^29^ and analyzed using python scripts to compute the correlation function defined by eq 2. Bayesian regression analysis was performed using the numpy,^30^ pandas,^31,32^ scipy^33^ and emcee^34^ packages. Plots were generated using matplotlib,^35^ seaborn,^36^ corner,^37^ and color maps from perfect-cmaps.^38^ Finally, structures were visualized and rendered for publication using Ovito.^39^

## Results

### Glass Transition and Kinetic Fragility

We begin by analyzing the temperature dependence of the molecular diffusivity (Figure 2a; also see Section S2). When obtaining these data from MD simulations we are limited by the time scale that is reachable via the latter. While one can reach on the order of 1 μs in total simulation length, the diffusivity rapidly decreases as the temperature is lowered. At lower temperatures, diffusion events become so infrequent that molecules appear immobilized on the MD time scale. Assuming a nearest neighbor distance of 3 Å and a simulation length of 10 ns, this implies that the diffusivity can no longer be reliably estimated if its value drops below approximately 9 Å^2^/10 ns ≈ 10^–14^ m^2^ s^–1^. For the present system, this is the case for temperatures below approximately 400 K (see Section S2 of the Supporting Information for an extended analysis). This is below the experimental melting point of pure perylene of around 550 K but above the experimental glass transition temperatures, which range around 250 K.^12^

**Figure 2:**
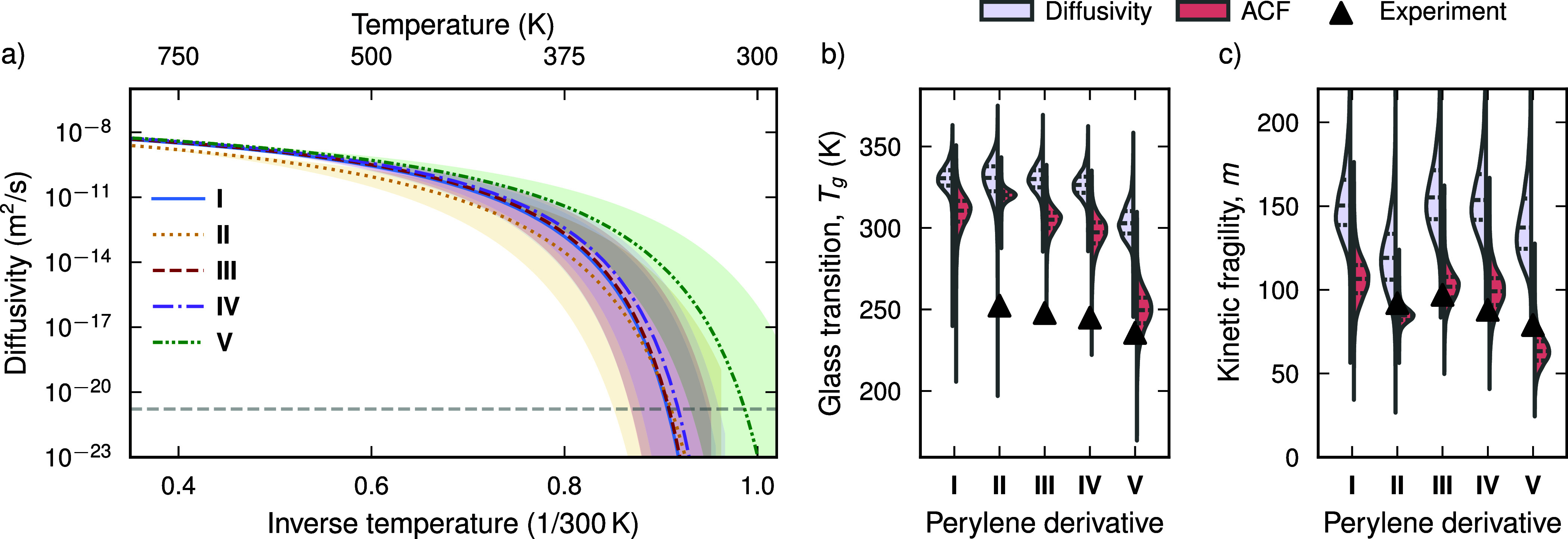
(a) Extrapolation of the temperature dependence of the Vogel–Fulcher–Tammann (VFT) fit of the diffusivity to lower temperatures (see Section S2 in the Supporting Information for the MD data). The glass transition temperature *T*_g_ is defined as the temperature where the mean squared displacement (MSD) over 100 s reaches 100 Å, denoted by the horizontal gray dashed line. The error band corresponds to one standard deviation. (b, c) Violin plots of (b) the glass transition temperature *T*_g_ and (c) the kinetic fragility *m* estimated from both the diffusivity and the normal vector ACF. Experimental values are from ref 12. *T*_g_ values were experimentally obtained from differential scanning calorimetry (DSC) first heating thermograms with a heating rate of 0.17 K·s^–1^. The kinetic fragility was obtained from fast scanning calorimetry (FSC) measurements for various cooling rates as *m* = −d log|*q*|/d­(*T*_g_/*T*_f_ *′*)|_*T*_f_ *′* =*T*_g__, where *q* is the cooling rate and *T*_f_ *′* is the fictive FSC temperature. The simulated values for the kinetic fragility were computed from the VFT parameters as *m* = *BT*_g_/[ln­(10)·(*T*_g_ – *T*_VF_)^2^)]. Note that *T*_g_ is typically not observed experimentally for derivative **I**, due to its strong tendency to crystallize.

In order to be able to gain information about the behavior at these temperatures, we need to extrapolate. However, since the diffusivity and other properties change rapidly over many orders of magnitude in this region, this extrapolation must be done with care and account for error propagation. To this end, we employ the VFT equation and combine it with Bayesian regression. The former describes the temperature dependence of, e.g., the viscosity or the diffusivity of fragile glass formers, allowing for non-Arrhenius behavior. While the VFT equation is empirical in nature, it is widely used in the analysis of glass-forming systems and provides an accurate fit for many experimental observations as well as the data obtained here (Figure 2a). For the diffusivity it reads

$$D\left(T\right)=D_{0}\exp\left[-B/\left(T-T_{\mathrm{VF}}\right)\right]$$ 4

where *D*_0_ is a prefactor, *T*_VF_ is the Vogel–Fulcher temperature, and *B* is a parameter akin to a pseudoactivation energy.^40^ The parameters of the VFT equation can in turn be used to compute the kinetic fragility *m* = *BT*_g_/[ln­(10) × (*T*_g_ – *T*_VF_)^2^)], where *T*_g_ is the estimated glass transition temperature.^41,42^

Due to the exponential in eq 4 extrapolation and error propagation require care, which we handle here via Bayesian regression. The latter is a technique in which a model *M*(**θ**) with parameters represented by a parameter vector **θ** = [*D*_0_, *T*_VF_, *B*] is fitted to a set of data D given prior information I , using Bayes’ theorem

p(θ|D,I)∝p(D|θ,I)p(θ|I) 5

The advantage of a Bayesian approach is 2-fold. First, prior beliefs are clearly stated in the prior distribution p(θ|I) . Second, error estimates are readily extractable from the posterior distribution p(θ|D,I) , since data uncertainties and errors can be encoded in the likelihood function p(D|θ,I) . We then sample the posterior distribution p(θ|D,I) via Markov-chain Monte Carlo (MCMC) simulations using the diffusivity data from MD simulations to fit the VFT equation (see Section S3 for details). This allows us to extrapolate the diffusivity to lower temperatures along with controlled error estimates (Figure 2a).

The temperature at which the system transitions into a glassy state is denoted by the glass transition temperature *T*_g_. *T*_g_ cannot be uniquely defined but is rather set by a pragmatic property-dependent threshold. For example, one often takes *T*_g_ as the temperature where the viscosity reaches a value of 10^11^ Pa·s.^42^ In the present work, when considering the diffusivity, we adopt a threshold of 17 × 10^–22^ m^2^/s, which corresponds to a MSD of 100 Å^2^ over 100 s. In other words, it specifies the onset of diffusion beyond the first-nearest neighbor shell. We emphasize that since the viscosity and similarly the diffusivity change very steeply around the glass transition (Figure 2a) the threshold value has only a modest effect on the values obtained for *T*_g_. For example, increasing or decreasing the threshold by 2 orders of magnitude changes our estimates for *T*_g_ by only ±5 K.

The glass transition temperatures obtained here are in semiquantitative agreement with experiments, and correctly predict the trend from **II** to **V** ^12^ (Figure 2b). However, the simulated *T*_g_ values are overestimated compared to experimental values obtained by differential scanning calorimetry (DSC) by 50–70 K. The predicted kinetic fragilities are also in agreement with experimentally obtained values from fast scanning calorimetry (FSC) (Figure 2c).

The glass transition temperature decreases systematically with increasing alkyl chain length. Conceptually, this can be explained by an increase in the effective volume available to each molecule caged by its neighbors, due to the longer pendant groups. It is, however, noteworthy that the kinetic fragility exhibits a maximum for **III**, which features a butyl pendant chain—a nontrivial behavior that is observed in both experiment and simulation.

### Revealing the Relaxation Processes

We now turn to the normal vector ACF *J*(τ) (eq 2) to gain further insight into the relaxation processes close to the glass transition (Figure 3a). We demonstrate the procedure for obtaining the temperature dependence of *J*(τ) for derivative **I**, noting that the general temperature dependence of *J*(τ) is consistent for all perylene derivatives **I–V** (see Supporting Information Section S4 for the ACFs for all perylene derivatives).

**Figure 3:**
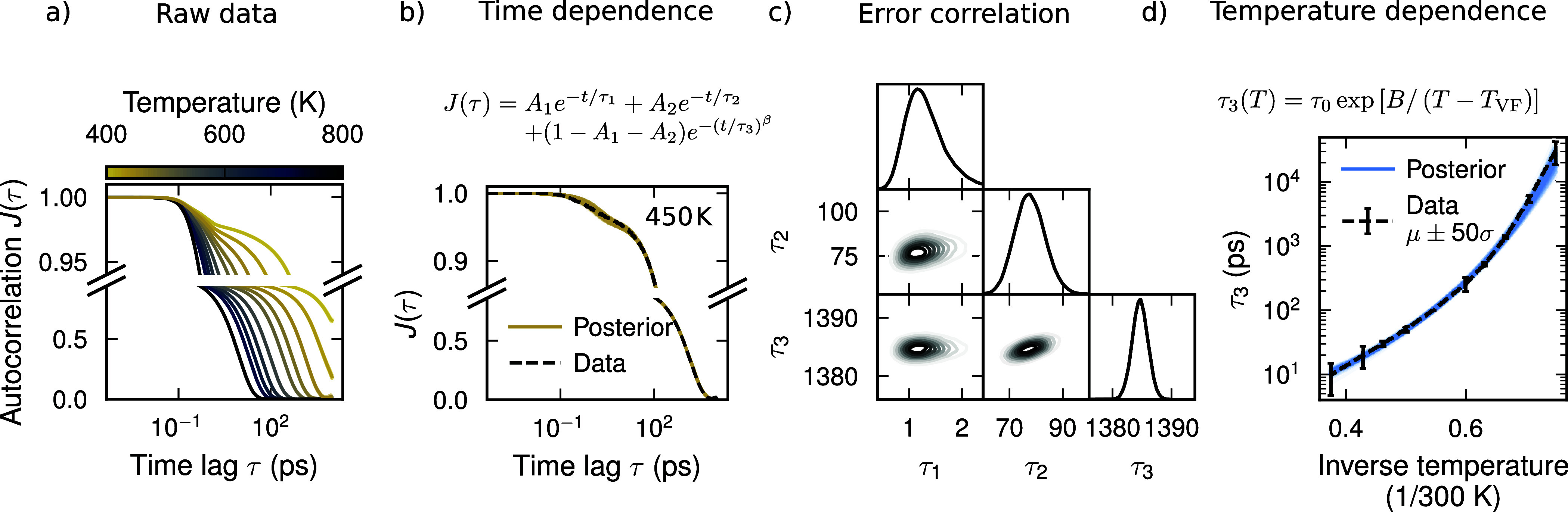
Bayesian regression workflow used to extrapolate the normal vector ACF to longer time scales. (a) Normal vector ACFs for perylene derivative **I** at different temperatures. Note that the *y*-axis has been split using different scales to reveal the multiple steps in the ACF. (b) Normal vector ACF for perylene derivative **I** at 45 K along with the corresponding posterior distribution of fits to eq 6. (c) Subset of the posterior distribution in (b), $p(\tau_{1},\tau_{2},\tau_{3}|D,I)$ , shown as a corner plot. (d) Fit to the VFT expression eq 7 using the mean μ, and standard deviation σ of the marginal distribution $p(\tau_{3}|D,I)$ .

First, we observe that the correlation time of *J*(τ) depends strongly on temperature, ranging from 100 ps at 800 K to >10 ns at 400 K. At 800 K the perylene molecules thus maintain their orientation over a time scale on the order of 100 ps, while they are effectively locked in their orientation over 10 ns at 400 K.

Second, the ACFs can be described by the sum of two exponential functions and one stretched exponential function, where the latter is a common feature of correlation functions in glassy systems^43^

$$J(\tau )=A_{1}e^{-\tau /\tau_{1}}+A_{2}e^{-\tau /\tau_{2}}+(1-A_{1}-A_{2})e^{(-\tau /\tau_{3}{)}^{\beta }}$$ 6

The time scales τ_1_, τ_2_, and τ_3_ are separated by several orders of magnitude at low temperatures, with τ_1_ ≈ 0.1 ps, τ_2_ ≈ 10 ps, and τ_3_ ≈ 1 ns at 450 K. β ≤ 1 is the stretch exponent for the long time scale component.

We can apply the same Bayesian regression workflow as for the diffusivity to estimate the glass transition temperature and kinetic fragility from the temperature dependence of the normal vector ACF. However, an additional step is required compared to the diffusivity, as the normal vector ACF needs to be fitted to eq 6 for each temperature (Figure 3b). Each fit yields a full posterior probability distribution $p(A_{1},A_{2},\tau_{1},\tau_{2},\tau_{3},\beta |T,D,I)$ . An estimate for the time scale of the slowest process captured by the ACF presented by τ_3_ with uncertainty estimates can then be obtained from the marginal distribution $p(\tau_{3}|T,D,I)$ for each temperature (Figure 3c). A VFT equation of the form

$$\tau_{3}(T)=\tau_{0}\exp[B/(T-T_{\mathrm{VF}})]$$ 7

is then fitted to the temperature dependence of τ_3_, which allows for a similar extrapolation to longer time scales as in the case of the diffusivity (Figure 4). Here, the threshold for τ_3_ above which the system is deemed to be in a glassy state was again taken to be 100 s.^42^ Note that the resulting glass transition temperature is relatively insensitive to this particular threshold, as increasing or decreasing the threshold by 2 orders of magnitude only changes *T*_g_ by ±7 K.

**Figure 4:**
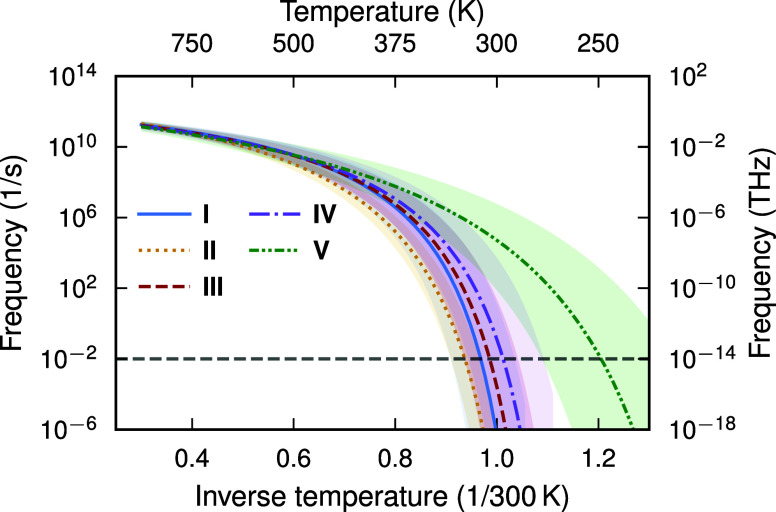
Extrapolation of the temperature dependence of the slowest process represented by τ_3_(*T*), to lower temperatures and thus lower frequencies. The error band corresponds to ± one standard deviation. *T*_g_ is defined to be the temperature which the time scale reaches 100 s, represented by the horizontal gray dashed line.

The estimates for both the glass transition temperature and the kinetic fragility from the normal-vector ACF and the diffusivity generally agree with each other (Figure 2b,c). Both schemes capture the trend of decreasing *T*_g_ with increasing length of the alkyl chain of the perylene derivative. However, the estimates from the diffusivity are higher than those from the normal-vector ACF typically by 10–30 K for the glass transition temperature and by 10–40 for the kinetic fragility. This difference is due to the two observables probing different processes. The diffusivity is sensitive to the diffusion of the monomers, while the normal vector ACF probes the rotational motion of the monomer. The normal vector ACF and the diffusivity are thus complementary. The difference in *T*_g_ between both observables suggests that the monomers continue to rotate on long time scales 10–30 K below the temperature at which diffusion has slowed down.

We can elucidate the relaxation processes in the system by decomposing the ACF into the contribution of each exponential function that make up *J*(τ) (Figure 5). The separation of time scales between the processes allows the selective application of frequency filters in the Fourier domain, corresponding to the time scales represented by τ_1_, τ_2_, and τ_3_. These filters are applied to the trajectory of a single perylene molecule extracted from the entire MD trajectory, and allows us to single out the dynamics that correspond to each process (see the supplementary movie for a visual representation of this scheme, and Section S5 of the Supporting Information for further details).

**Figure 5:**
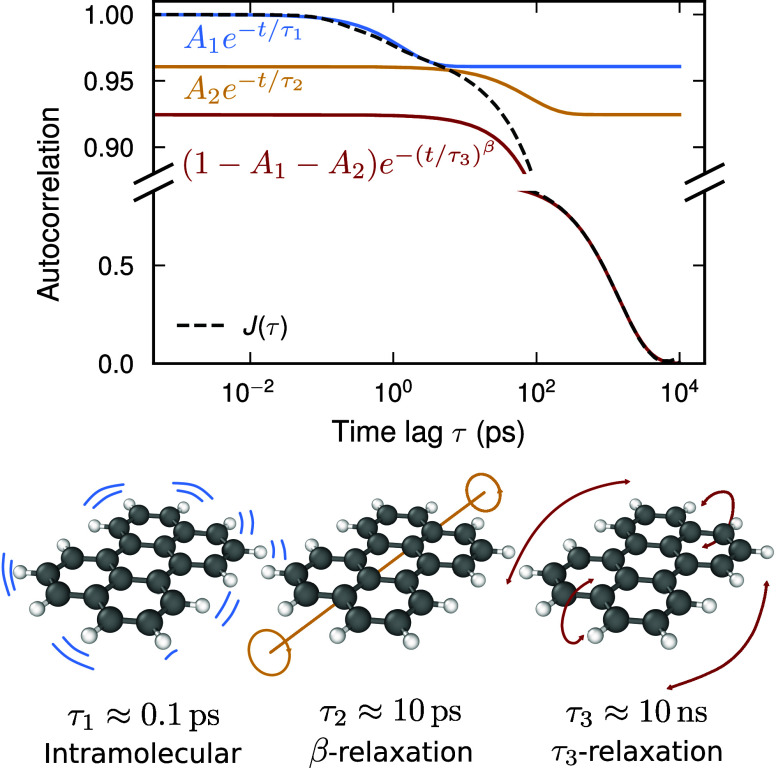
Decomposition of the normal vector ACF *J*(τ) into individual exponential functions representing three different relaxation processes. The fastest process with a correlation time of about 0.1 ps at 450 K corresponds to intramolecular atomic motion. The second one (β-relaxation) with a correlation time of approximately 10 ps at 450 K corresponds to librational motion and twisting of the perylene core. Finally, the slowest process (τ_3_-relaxation) with a correlation time of approximately 10 ns at 450 K corresponds to the hindered rotational reorientation of the perylene molecules due to intermolecular interactions.

We study the dynamics of perylene derivative **I** at a temperature of 450 K as an example of this scheme (Figure 5). The fastest process with time scale τ_1_ corresponds to intramolecular atomic motion. The second fastest process, τ_2_, corresponds to β-relaxation enforced by caging by neighboring molecules, such as libration and twisting of the perylene core. Neither the τ_1_ nor the τ_2_ processes significantly affect the orientation of the molecule, as is evident by their small amplitude. The bulk of the autocorrelation function *J*(τ) is made up of the slow τ_3_ process. τ_3_ corresponds to cooperative intermolecular processes, such as reorientation of molecules. The reorientation of a molecule requires neighboring molecules to rotate, which takes place over rapidly increasing time scales as the temperature is decreased. The experimental and simulated values of the kinetic fragility indicate that all derivatives studied in this work are fragile glass formers. In fragile glass formers, the level of cooperation decreases significantly at temperatures greater than *T*_g_.^44^ That τ_3_ captures cooperative reorientation even in the supercooled regime at 450 K thus indicates that it is sensitive to processes that are more prominent close to the glass transition in fragile glass formers. Based on this, we attribute τ_3_ to be related to α-relaxation, and that the microscopic mechanism driving glass formation in perylene derivatives **I–V** is the cooperative reorientation of the molecules.

## Discussion

Given the sources of uncertainty related to the underlying empirical force field used in the MD simulations and the extrapolation over many orders of magnitude, we consider the overall agreement of the predicted glass transition temperatures and kinetic fragilities with the experimental data very encouraging. The normal vector ACF in particular show semiquantiative agreement with experiments, with the ACF systematically yielding both lower glass transition temperatures and kinetic fragilities than the diffusivity (Figure 2b,c). This difference highlights the complementarity of the diffusivity and the normal vector ACF, as they are more sensitive to molecular diffusion and rotation, respectively. The estimated higher value of the glass transition temperature from the diffusivity can be understood as molecular diffusion freezing in at a higher temperature compared to rotation. The processes driving glass formation are thus cooperative rotational processes, as elucidated by the decomposition of the normal vector ACF. This is supported by the large kinetic fragility deduced for all derivatives (Figure 5). Capturing both diffusion and rotation is hence key in order to accurately describe the relaxation processes in the fragile perylene derivatives studied in this work.

Both the normal vector ACF and the diffusivity systematically overestimate the glass transition temperature and the kinetic fragility compared to experiment. The overestimation of the kinetic fragility suggests that the processes represented by τ_3_ in the MD simulation are slower than those encountered during experiments. This could be caused by the intermolecular interactions in the simulation being somewhat too soft, which would point toward a limitation in the accuracy of the underlying force field. Another possible explanation could be that the normal vector ACF overestimates the time scale of processes in the system. Third, the VFT equation is an empirical model, and extrapolating to lower temperatures may introduce a systematic error.^45^ In order to go beyond empiricism, more explicit characterizations of the glass dynamics, e.g. mode coupling theory, could be pursued.^46,47^

As noted in the introduction, experimentally the glass transition can also be detected as a change in the thermal expansion of the material, an approach that is also occasionally adopted in simulations.^16,48–53^ It is therefore instructive to contrast this approach with the one based on diffusivity and time ACFs used in the present work. For the present systems we observe a change in the thermal expansion coefficient at a temperature of around 400 K, which would suggest much higher glass transition temperatures (Section S6). At the same time, one can observe from the analysis of the normal vector ACF that in this temperature range the relaxation time for the slowest process τ_3_ reaches the limit of the MD time scale. Similar limitations of, for example, the diffusivity on MD time scales compared to experiments have also been observed for other conjugated systems.^54^ The change in the thermal expansion is thus merely a direct result of the limited MD time scale rather than a feature of the system. As a result, at least for the present systems the analysis of the thermal expansion cannot be expected to yield a physically meaningful estimate of the glass transition temperature. Further comparison between the thermal expansion method and the method presented in the present work is needed to fully characterize in which situations they are applicable.

## Conclusions

The method for extending the temperature range using Bayesian regression presented in this work allows us to study relaxation processes in liquid and supercooled liquid systems containing hundreds to thousands of molecules. Hence, it is possible to make material-specific predictions for the glass transition temperature and the kinetic fragility. The general approach is directly extendable to other systems, where especially the diffusivity can be readily computed. This work serves as a first step toward accurately describing the complex relaxation processes in multicomponent mixtures of perylene derivatives. Insight into these relaxation processes is key in obtaining a systematic understanding of the dynamics of perylene derivatives, enabling the design of stronger and more stable glass forming system.

## Supplementary Material





## Notes

Calculation of the normal vector autocorrelation function; extracting diffusivity from the mean-squared displacement; details on Bayesian fitting; calculated autocorrelation functions for all perylene derivatives studied in this work; decomposing the autocorrelation function using Fourier filtering; supplementary video of the dynamics from decomposing the autocorrelation function; estimating glass transition tempeatures from simulated annealing; and traces and corner plots from MCMC sampling (PDF)
Movie: Relaxation process separation (GIF)
