# Supplementary material for: Probing Glass Formation in Perylene Derivatives via Atomic-Scale Simulations and Bayesian Regression
Source: J Phys Chem B. 2025 Jun 23;129(26):6613–9. doi: 10.1021/acs.jpcb.5c00837 (PMC12235637; doi:10.1021/acs.jpcb.5c00837)
Supplement: Supplementary file 1 [file jp5c00837_si_001.pdf]

Supporting Information

Probing Glass Formation in Perylene Derivatives via Atomic Scale Simulations and Bayesian Regression

Eric Lindgren[1], Jan Swenson[1], Christian Müller[2], and Paul Erhart[1,*]

[1] *Department of Physics, Chalmers University of Technology, SE-412 96 Gothenburg, Sweden*
[1] *Department of Chemistry and Chemical Technology, Chalmers University of Technology, SE-412 96 Gothenburg, Sweden*
[*] *erhart@chalmers.se*

Contents

S1 Computing the normal vector autocorrelation function **S2**

S2 Extracting the diffusivity from the mean-squared displacement **S2**

S3 Bayesian fitting **S4**
S3.1 Fitting the autocorrelation function to triple exponential S4
S3.2 Fitting the diffusivity and autocorrelation to the VFT equation S5

S4 Autocorrelation functions for all perylene derivatives **S6**

S5 Decomposing the autocorrelation function **S7**

S6 Estimating the glass transition temperature from simulated annealing **S7**

S7 MCMC sampling **S7**
S7.1 MCMC traces: Fitting the diffusivity S7
S7.2 MCMC traces: Fitting the VFT equation to the diffusivity S8
S7.3 MCMC traces: Fitting the normal vector ACF S9
S7.4 MCMC traces: Fitting the VFT equation to the normal vector ACF S10

S8 Corner plots **S11**
S8.1 Corner plots: Fitting the diffusivity S11
S8.2 Corner plots: Fitting the VFT equation to the diffusivity S12
S8.3 Corner plots: Fitting the normal vector ACF S13
S8.4 Corner plots: Fitting the VFT equation to the normal vector ACF S14

References **S16**

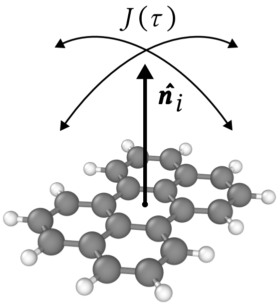

Figure S1: Schematic representation of the normal vector autocorrelation function $J(\tau)$, see Eq. (S3).

S1 Computing the normal vector autocorrelation function

Given $\boldsymbol{r}_i^m(t)$ as the position of atom m in molecule i at time t, the centroid position of molecule i with M atoms is defined as

$$\boldsymbol{r}_i(t) = \frac{1}{M} \sum_{m=1}^{M} \boldsymbol{r}_i^m(t). \tag{S1}$$

We can now define the normal vector correlation function,

$$C_{\hat{n}\hat{n}}(r,\tau) = \left\langle \sum_{i=1}^{N} \sum_{j=1}^{N} \delta(\boldsymbol{r} - \boldsymbol{r}_{ij}) \hat{\boldsymbol{n}}_i(t) \cdot \hat{\boldsymbol{n}}_j(t+\tau) \right\rangle_t , \tag{S2}$$

where $\hat{\boldsymbol{n}}_i(t)$ is the normal vector of molecule i, and is calculated by computing the normal to the point cloud of atoms in the molecule, $\{\boldsymbol{r}_i^m(t)\}$. The ensemble average is taken over all frames in the trajectory. In particular, we study the special case of the normal vector autocorrelation function $C_{\hat{n}\hat{n}}(0,\tau)$, denoted $J(\tau)$.

$$J(\tau) = C_{\hat{n}\hat{n}}(\tau) = \langle \hat{\boldsymbol{n}}_i(t) \cdot \hat{\boldsymbol{n}}_i(t+\tau) \rangle_{it} , \tag{S3}$$

with the ensemble average taken over all frames t and each molecule i in the system. Equation (S3) can be efficiently computed from the signal $\hat{\boldsymbol{n}}_i(t)$ using the Wiener-Kinchin theorem.

We may also extract the standard error as an uncertainty estimate for $J(\tau)$ from the correlation function for each molecule $J_i(\tau)$ before computing the ensemble average in equation Eq. S3, using the central limit theorem,

$$\sigma_J(\tau) = \sqrt{\mathrm{Var}\left(\{J_i\}_{i=1}^{N}\right)} / \sqrt{N}, \tag{S4}$$

where N is the number of molecules in the system. The normal vector $\hat{n}_i(t)$ and $J(\tau)$ are schematically represented in Fig. S1.

Two production molecular dynamics (MD) simulations, with a length of 100 ps and 10 ns respectively, were run in order to get the same number of statistics for short and long time lags. The trajectory files were written every 1 fs for the 100 ps simulation and every 100 fs for the 10 ns simulation. The normal vector autocorrelation functions (ACFs) according to Eq. S3 were calculated for both production runs and then spliced together at a time lag of $\tau = 1$ ps. This splicing was also done for the standard error in equation Eq. S4.

S2 Extracting the diffusivity from the mean-squared displacement

We obtained the molecular diffusivity D for each perylene derivative **I**–**V** and temperature, from the mean squared displacement (MSD) $\langle \Delta r^2 \rangle$ of the molecular centroid positions[1],

$$\langle \Delta r^2 \rangle = 6D\tau. \tag{S5}$$

The diffusivity was computed using production runs with a duration of up to 10 ns. D was extracted by performing a linear fit using Bayesian regression to the MSD (Fig. S2). Note that D for

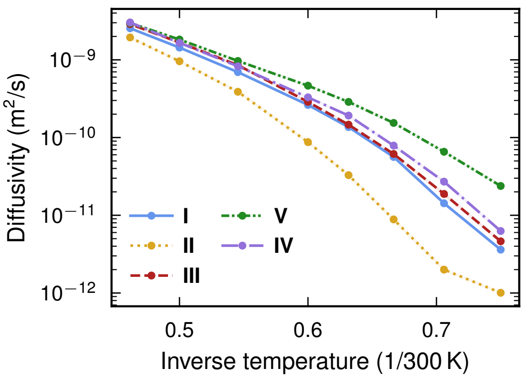

Figure S2: Diffusivity computed from the MSD for each perylene derivative **I**–**V**

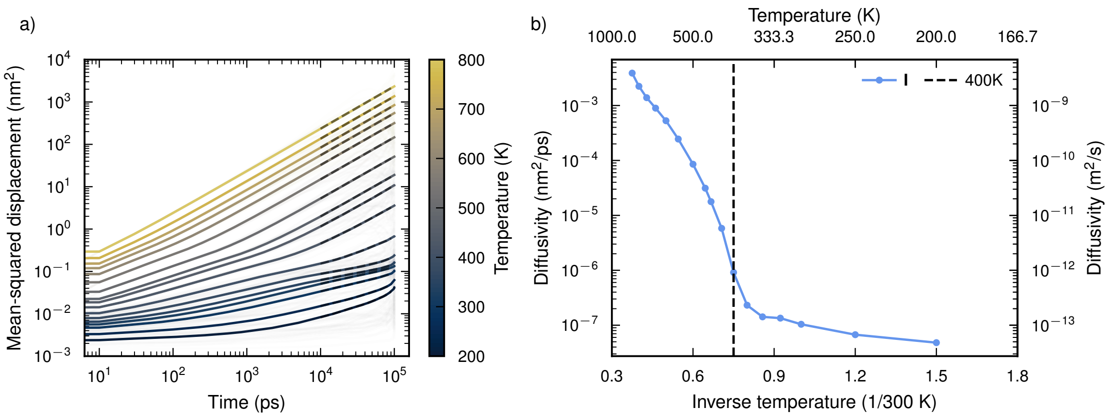

Figure S3: a) Mean-squared displacement and b) diffusivity obtained from the MSD measured over 100 ns MD simulations, as a function of temperature for perylene derivative **I**. Note that the diffusivity exhibits a discontinuity at 400 K, which is due to the diffusion processes reaching the timescale of the MD simulation.

high temperatures change slope; this is due to some of the derivatives transitioning to a gaseous phase. When fitting the Vogel-Fulcher-Tammann (VFT) equation we thus only considered D for temperatures < 700 K.

The lower bound in temperature for the diffusivity calculations was 400 K, due to the diffusion processes slowing down to the timescale of the MD simulation at this temperature. To demonstrate this effect, we performed a set of 100 ns MD simulations for perylene derivative **I** for a broad range of temperatures, and calculated the diffusivity (Fig. S3). Specifically, for perylene derivative **I** at 400 K, the diffusivity is 1×10^{-6} nm ps^{-1}. Over the course of a 100 ns MD simulation, this means that on average each perylene molecule moves a distance of $\approx$3 Å. This distance is comparable to the nearest-neighbor distance between molecules, which means that at 400 K a perylene molecules barely has time to move out of the shell formed by it's neighbors.

For temperatures lower than 400 K the diffusivity decreases further, and the average distance that a perylene molecule moves becomes smaller than the nearest neighbor distance. This means that on the timescale of the MD simulation, the molecules will appear to be locked in their nearest-neighbor cages, and we can no longer reliable estimate the diffusivity. We can approximate the lowest diffusivity we can estimate based on the MD simulation time using the nearest-neighbor distance. Assuming a nearest-neighbor distance of R, we require that $\langle \Delta r^2 \rangle > R^2$. This implies $R^2 > 6DT$ where T is the total simulation time, from Eq. S5. By assuming a nearest neighbor distance R of 3 Å and a simulation time T of 100 ns, we obtain $D \gtrsim 10^{-13}$ m^2 s^{-1}. If the diffusivity drops below this threshold it can no longer be reliably estimated, which for the present system occurs at approximately 400 K.

S3 Bayesian fitting

In this work, we use a Gaussian likelihood function, which under the assumption of independent and identically distributed N_d data $d_i \in \mathcal{D}$, here takes the form,

$$p(\mathcal{D}|\boldsymbol{\theta}, \mathcal{I}) = \prod_{i=1}^{N_d} \frac{1}{\sqrt{2\pi\sigma_i^2}} e^{-(M(\boldsymbol{\theta})-d_i)^2/(2\sigma_i^2)}, \tag{S6}$$

with σ_i^2 as the variance of the residual for datum d_i. We use σ_i to encode the standard error of the diffusivity and the ACF, as well as to perform error propagation.

S3.1 Fitting the autocorrelation function to triple exponential

Let $J(\tau)_T$ denote the ACF for temperature T. For each perylene derivative a–e, we have a data set of ACFs $\mathcal{D} = \{J(\tau)_{T_i}\}_{i=1}^{L}$, where L is the number of distinct temperatures studied for this derivative. In this stage, we used a Gaussian likelihood on the form in equation Eq. S6, where we let $\sigma_i = \sigma/\sigma_{J(\tau)_{T_i}}$, with $\sigma_{J(\tau)_{T_i}}$ being the standard error for ACF $J(\tau)_{T_i}$ at temperature T_i, and σ is a free parameter. This is a heteroscedastic error model, in which each datum has an individual error. In total, the set of free parameters to optimise in this stage was $\boldsymbol{\theta} = \{A_1, A_2, \tau_1, \tau_2, \tau_3, \beta, \sigma\}$.

The priors for each of the parameters were set as follows,

$$p(A_i|\mathcal{I}) = \frac{1}{0.2} e^{-|A_i/0.1|}, \text{ for } A_1, A_2 \tag{S7}$$

$$p(\tau_1|\mathcal{I}) = \frac{\sqrt{2}}{5\sqrt{\pi}} e^{-(\tau_1/5)^2/2}, x > 0 \tag{S8}$$

$$p(\tau_2|\mathcal{I}) = \frac{1}{50\sqrt{2\pi}} e^{-((\tau_2-10)/50)^2/2} \tag{S9}$$

$$p(\tau_3|\mathcal{I}) = \frac{1}{\sqrt{2\pi}} e^{-(\log_{10}(\tau_3)-3)^2/2} \tag{S10}$$

$$p(\beta|\mathcal{I}) = \frac{\sqrt{2}}{0.1\sqrt{\pi}} e^{-((x-1)/0.1)^2/2}, x = 2 - \beta \tag{S11}$$

$$p(\sigma|\mathcal{I}) = \frac{\sqrt{2}}{\sqrt{\pi}} e^{-\sigma^2/2}, x > 0 \tag{S12}$$

with the same priors being used for all temperatures. Note that the prior for τ_3 is for $\log_{10}(\tau_3)$, due to τ_3 spanning several orders of magnitude throughout the temperature range. The joint prior then becomes

$$\begin{aligned} p(\boldsymbol{\theta}|\mathcal{I}) = & p(A_1|\mathcal{I})p(A_2|\mathcal{I}) \\ & p(\tau_1|\mathcal{I})p(\tau_2|\mathcal{I})p(\tau_3|\mathcal{I}) \\ & p(\beta|\mathcal{I})p(\sigma|\mathcal{I}). \end{aligned} \tag{S13}$$

We then used Markov-chain Monte Carlo (MCMC) sampling to sample the posterior $p(\boldsymbol{\theta}|\mathcal{D}, \mathcal{I})$. For numerical stability, the likelihood and priors were rewritten as the log-likelihood and log-prior respectively. Optimizing the posterior or the log-posterior does not change the resulting distributions, and thus the quantity that was optimized was, up to a constant,

$$\ln(p(\boldsymbol{\theta}|\mathcal{D}, \mathcal{I}, T)) = \ln(p(\mathcal{D}|\boldsymbol{\theta}, \mathcal{I}, T)) + \ln(p(\boldsymbol{\theta}|\mathcal{I})). \tag{S14}$$

See Sect. S7 and Sect. S8 in the Supplementary Information for traces and corner plots resulting from the Bayesian fitting procedure.

$$J(\tau) = A_1 e^{-\tau/\tau_1} + A_2 e^{-\tau/\tau_2} + (1 - A_1 - A_2) e^{(-\tau/\tau_3)^\beta}, \tag{S15}$$

The triple-exponential model in equation Eq. S15 has six free parameters, and combined with the noise parameter σ we thus obtain a seven-dimensional posterior distribution $p(\boldsymbol{\theta}|\mathcal{D}, \mathcal{I}, T)$. From the posterior distribution, the marginal distribution for τ_3 can then be extracted,

$$p(\tau_3|\mathcal{D}, \mathcal{I}, T) \propto \int p(\boldsymbol{\theta}|\mathcal{D}, \mathcal{I}, T) dA_1 dA_2 d\tau_1 d\tau_2 d\beta d\sigma. \tag{S16}$$

Computationally, the marginal distribution can be extracted by only studying the samples distributions for the marginal parameters of interest. Mean $\mu_{\tau_3}(T)$ and standard deviation $\sigma_{\tau_3}(T)$ of $\tau_3(T)$ were then extracted from $p(\tau_3|\mathcal{D},\mathcal{I},T)$ in order to inform the second stage of the regression process.

S3.2 Fitting the diffusivity and autocorrelation to the VFT equation

Both the diffusivity and τ_3 were fit to a VFT equation using the same framework, as described in this section. The only difference between the two is the sign in the exponential of the VFT equation, which was negative for the diffusivity since the diffusivity decreases with temperature. We present the fitting for τ_3 here as an example.

We fitted a VFT equation the mean values $\tau_3(T)$, $\mu_{\tau_3}(T)$, from the first stage of the process,

$$K(T;\boldsymbol{\theta'}) = \tau_3^0 \exp \frac{B}{k_B(T - T_{\text{VF}})}, \tag{S17}$$

where k_B is the Boltzmann constant. The VFT equation is an experimentally observed law that the non-Arrhenius behavior of α-relaxation in glass forming systems obeys, where τ_3^0, B, and T_{VF} are empirical fitting parameters[2].

Note that the VFT equation as written in the main paper does not include k_B, and is instead written on the common form $K(T;\boldsymbol{\theta'}) = \tau_3^0 \exp \frac{B}{(T-T_{\text{VF}})}$ However, when actually fitting the VFT equation we introduced k_B for numerical reasons as this yields a value of $B \leq 1$. k_B was then absorbed back into B for all subsequent calculations of T_g, m etc.

The data set consisted of $\mathcal{D}' = \{\mu_{\tau_3}(T_i)\}_{i=1}^{L}$. In this case also a Gaussian likelihood with heteroscedastic errors $\sigma_i = \sigma'/\sigma_{\tau_3}(T)$ was used, with $\sigma_{\tau_3}(T)$ as the estimated standard deviation of the posterior distribution for τ_3. In total, four free parameters were fitted at this stage, $\boldsymbol{\theta'} = \{\tau_3^0, B, T_{\text{VF}}, \sigma'\}$.

The priors used in the second stage of the fitting procedure were,

$$p(\tau_3^0|\mathcal{I}) = \frac{\sqrt{2}}{\sqrt{\pi}} e^{-(\tau_3^0)^2/2}, x > 0 \tag{S18}$$

$$p(B|\mathcal{I}) = \frac{\sqrt{2}}{\sqrt{\pi}} e^{-B^2/2}, x > 0 \tag{S19}$$

$$p(T_{\text{VF}}|\mathcal{I}) = \frac{1}{200\sqrt{2\pi}} e^{-(T_{\text{VF}}-200)/200)^2/2} \tag{S20}$$

$$p(\sigma'|\mathcal{I}) = \frac{\sqrt{2}}{\sqrt{\pi}} e^{-\sigma'^2/2}, x > 0 \tag{S21}$$

The log-posterior was then optimized using MCMC-sampling, similarly to the first stage of the regression process. See Sect. S7 and Sect. S8 in the Supplementary Information for all traces and corner plots resulting from the Bayesian fitting procedure.

The resulting posterior distribution $p(\boldsymbol{\theta'}|\mathcal{D}',\mathcal{I}')$ of the fit to the VFT equation was then used to extrapolate τ_3 to lower temperatures, feeding the posterior distribution samples through the model in equation Eq. S17. The glass transition temperature T_g is roughly taken to be the temperature at which the slowest relaxation process in the system exceeds 100 s[2]. We therefore took the temperature at which $\tau_3(T)$ exceeded 100 s for each sample of the posterior distribution as an individual estimate of T_g. In total, for each molecule a–e, we obtained a distribution $p(T_g|\mathcal{D},\mathcal{D}',\mathcal{I})$ of estimates of the glass transition temperature T_g. Note that the estimate for T_g is relatively stable with regard to the choice of cutoff time for $\tau_3(T)$. Decreasing the time to 1 s or increasing it to 10 000 s changes T_g by 7 K and −5 K, respectively. These changes are smaller than the standard deviation of $p(T_g|\mathcal{D},\mathcal{D}',\mathcal{I})$, which typically is approximately 10 K to 20 K for the systems under study.

The glass transition temperature was similarly estimated from the diffusivity $D(T)$ as obtained from the MSD, using the same workflow. In this case, the inverse of the diffusivity was fitted against a VFT equation. The MSD over 100 s was then calculated as a function of temperature, and T_g was estimated as the temperature at which the MSD reaches 100 Å^2.

S4 Autocorrelation functions for all perylene derivatives

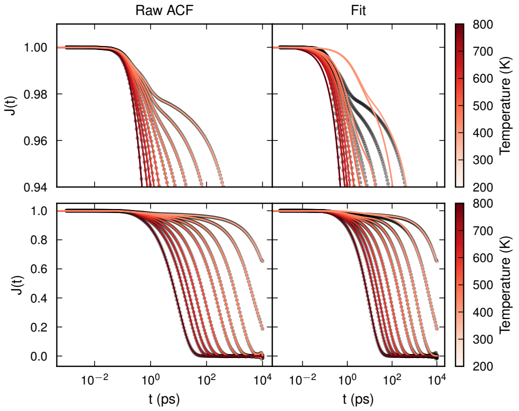

(a) Normal vector ACF for derivative **I**

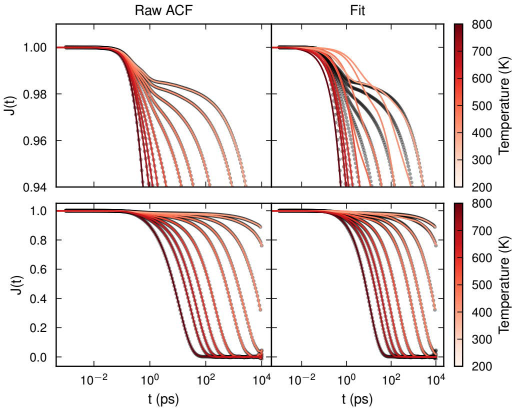

(b) Normal vector ACF for derivative **II**

Figure S4: Calculated and fitted ACFs using (S15). Error bands of the fits are plus and minus one standard deviation.

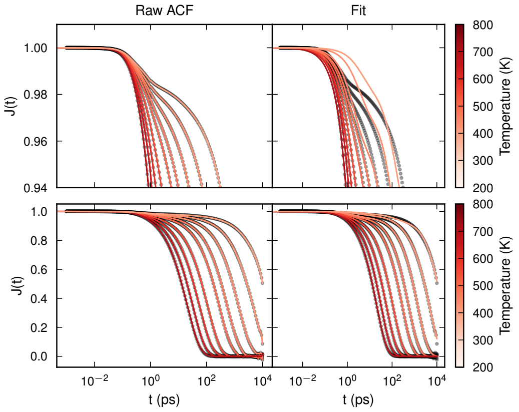

(a) Normal vector ACF for derivative **III**

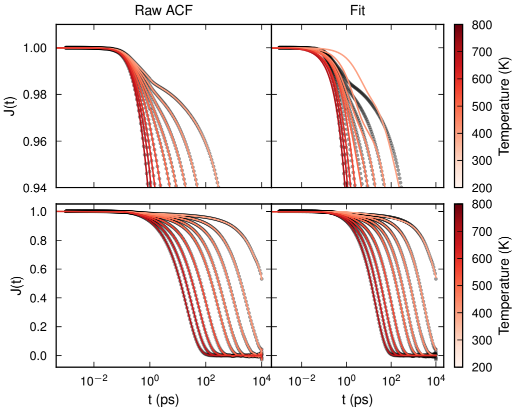

(b) Normal vector ACF for derivative **IV**

Figure S5: Calculated and fitted ACFs using (S15). Error bands of the fits are plus and minus one standard deviation.

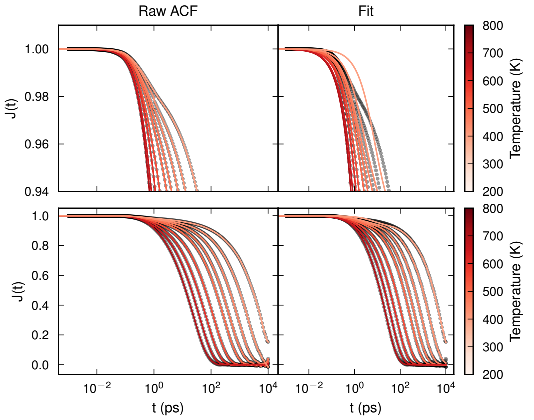

Figure S6: Calculated and fitted ACFs using (S15). Normal vector ACF for derivative **IV**

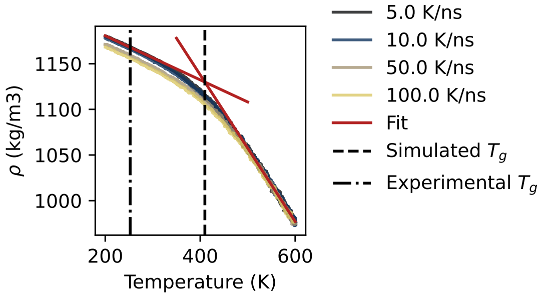

Figure S7: Glass transition temperature T_g estimated via simulated annealing using four different cooling rates for ethyl-perylene, a perylene derivative denoted II in this work. T_g is taken as the point at which the slope of the density changes, roughly at 412 K. This estimate from simulated annealing is severely overestimated compared to the experimentally measured glass transition temperature of 252.35 K from[3]. Note that the system size here is significantly larger than in the main paper, consisting of 5179 residues for a total of 217 322 atoms.

S5 Decomposing the autocorrelation function

Let $\{\boldsymbol{r}_i^k(t)\}_{k=1}^M$ be a trajectory of the coordinates of the atoms in a specific molecule i. The trajectory of a single molecule i was extracted and the centroid of the molecule, $\boldsymbol{r}_i(t)$, was placed in the middle of the simulation cell for each frame to avoid effects of the molecule moving over the periodic boundaries. The power spectrum of for each Cartesian component of the shifted trajectory was then computed, convoluted with a filter $F(\omega)$ in the Fourier domain, and the filtered power spectrum $S'(\omega)$ was then back transformed to yield a filtered trajectory $\{\boldsymbol{r}_i^{'k}(t)\}_{k=1}^M$,

$$\begin{aligned} S_l(\omega) &= \mathcal{F}\left[r_{il}^k(t)\right]^2(\omega), l \in \{x, y, z\} \\ S_l'(\omega) &= S_l(\omega) * F(\omega) \\ \rightarrow r_{il}^{'k}(t) &= \mathcal{F}^{-1}\left[S_l'(\omega)\right](t) \end{aligned} \tag{S22}$$

where $\mathcal{F}$ denotes the Fourier transform. The filter $F(\omega)$ is a simplified bandpass filter, on the form

$$F(\omega) \begin{cases} 1, & \omega_1 \leq \omega \leq \omega_2 \\ 0, & \text{otherwise.} \end{cases} \tag{S23}$$

By setting the filter frequencies ω_1 and ω_2 to match the expected time scales for τ_1, τ_2 and τ_3, this scheme gives a rough decomposition into what types of motion take place on the different timescales and allows us to somewhat elucidate what processes the ACF capture. A visualization of the decomposition scheme is available in the supplementary movie, Video V1.

S6 Estimating the glass transition temperature from simulated annealing

Here we demonstrate the use of simulated annealing for predicting the glass transition temperature of derivative **II** Fig. S7. Following the general approach set out in the literature, T_g is taken as the point at which the density changes slope[4–9]. This approach yields T_g that is overestimated by approximately 150 K, compared to experiments using differential scanning calorimetry from[3].

S7 MCMC sampling

Note that all the traces presented in this section have been sub-sampled by the maximum autocorrelation time over all chains. The traces thus only contain uncorrelated values.

S7.1 MCMC traces: Fitting the diffusivity

In the interest of space, we only show a representative trace plot for derivative **I** at 450 K (Fig. S8). All traces for the remianing perylene derivatives and temperatures are similar.

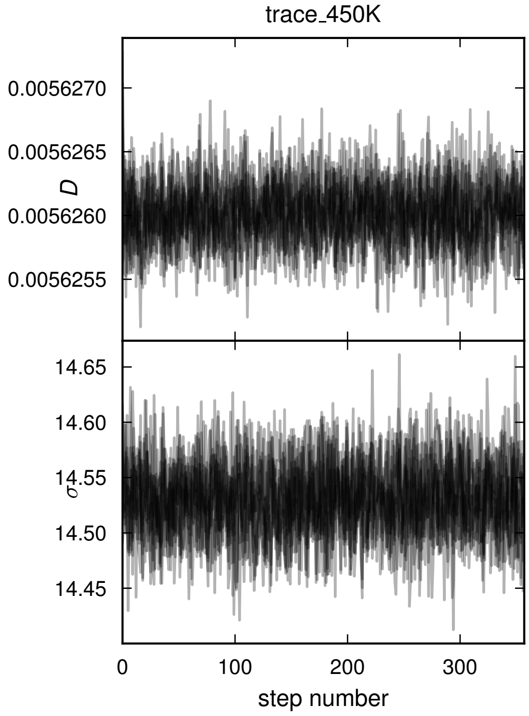

Figure S8: MCMC parameter trace for fitting the diffusivity in equation (S5) to the MSD at 450 K for derivative **I**

S7.2 MCMC traces: Fitting the VFT equation to the diffusivity

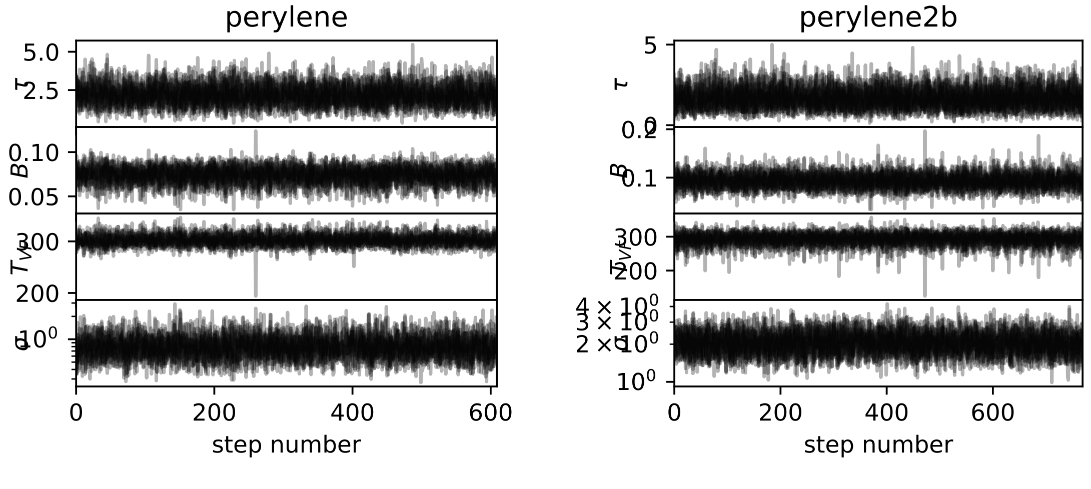

(a) Derivative **I**

(b) Derivative **II**

Figure S9: MCMC parameter trace for the fit of the VFT equation to the diffusivity.

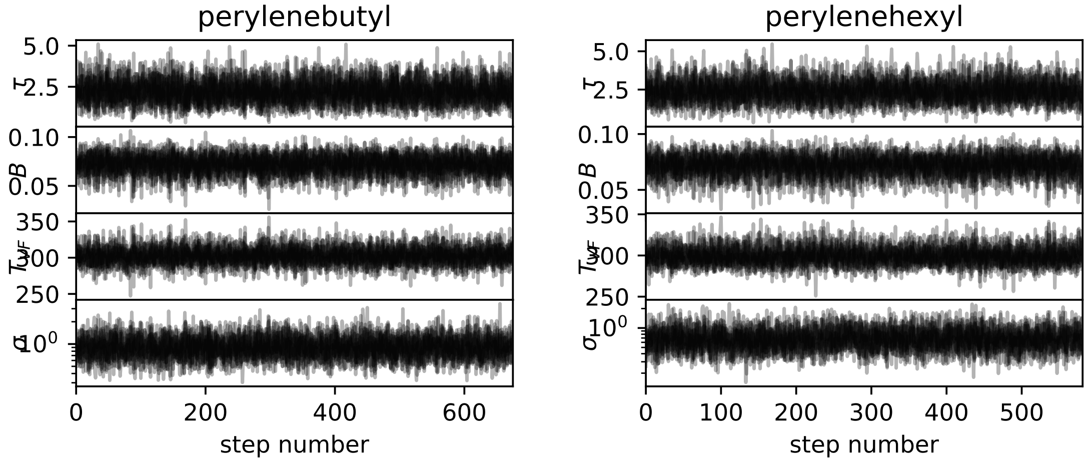

(a) Derivative **III**

(b) Derivative **IV**

Figure S10: MCMC parameter trace for the fit of the VFT equation to the diffusivity.

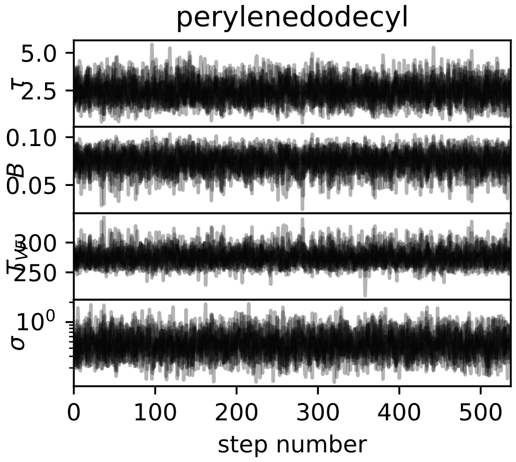

Figure S11: MCMC parameter trace for the fit of the VFT equation to the diffusivity for derivative **V**

S7.3 MCMC traces: Fitting the normal vector ACF

In the interest of space, we only show a representative trace plot for derivative **I** at 450 K (Fig. S12). All traces for the remianing perylene derivatives and temperatures are similar.

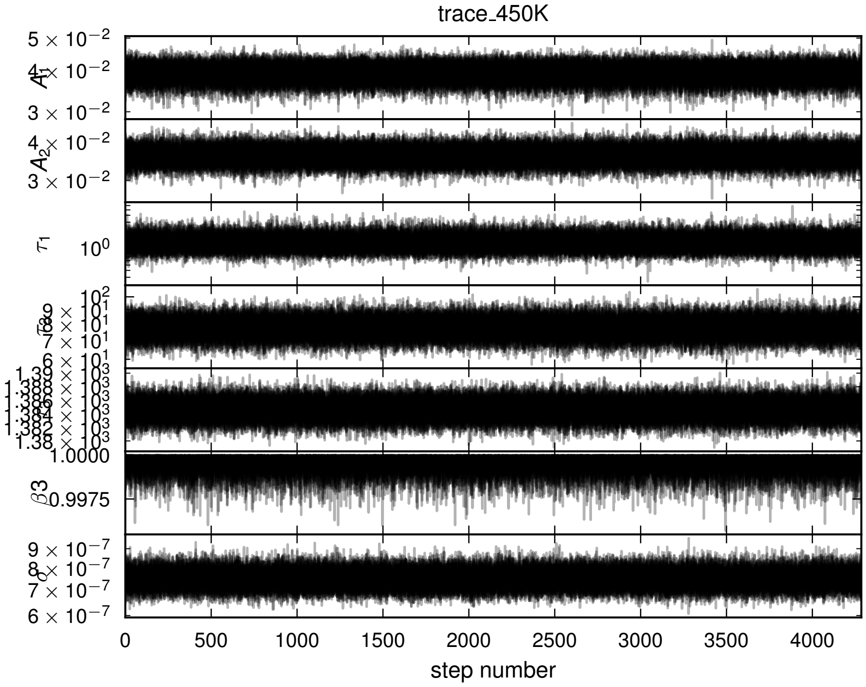

Figure S12: MCMC parameter trace for fitting the triple exponential function in equation (S15) to the normal vector ACF at 450 K for derivative **I**

S7.4 MCMC traces: Fitting the VFT equation to the normal vector ACF

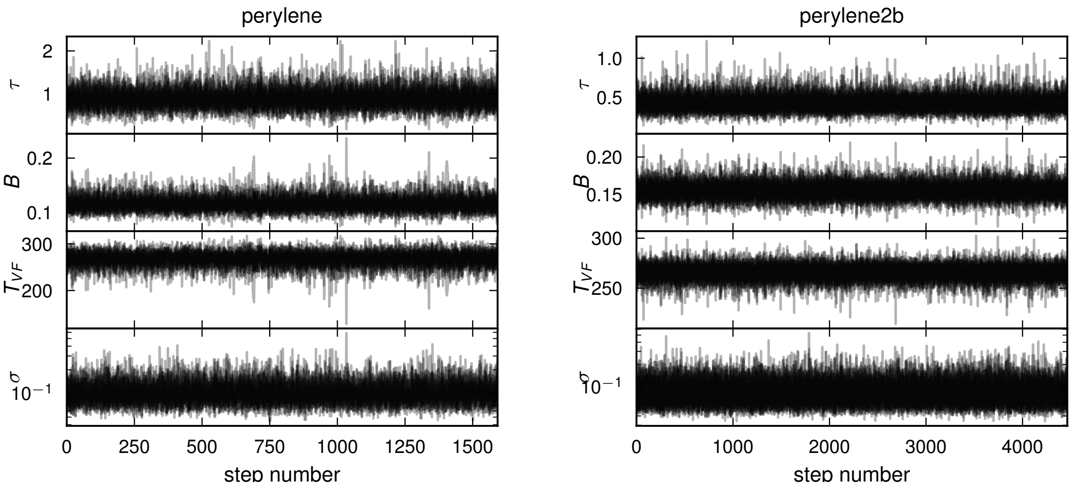

(a) Derivative **I**

(b) Derivative **II**

Figure S13: MCMC parameter trace for the fit of the VFT equation to the normal vector ACF.

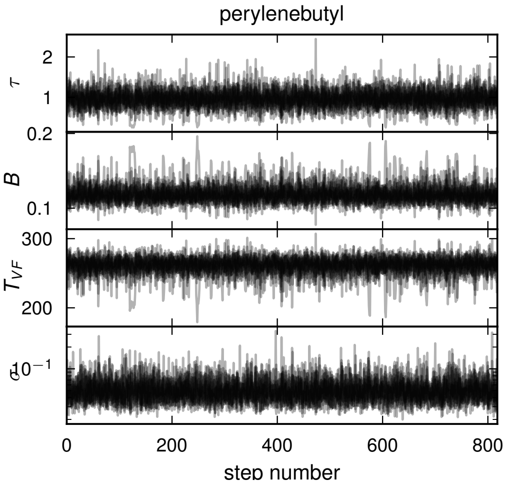

(a) Derivative **III**

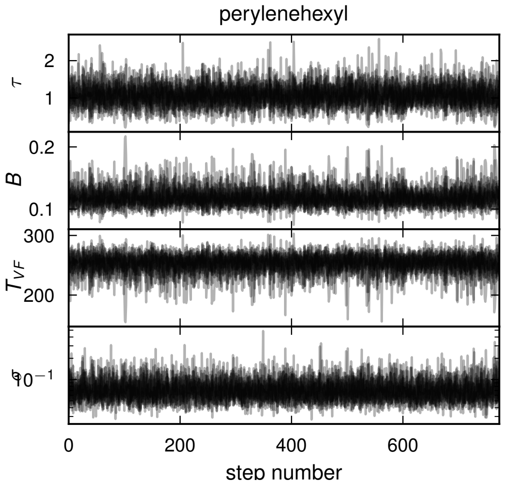

(b) Derivative **IV**

Figure S14: MCMC parameter trace for the fit of the VFT equation to the normal vector ACF.

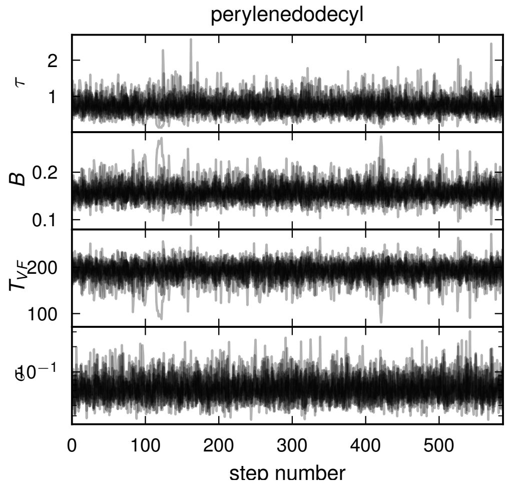

Figure S15: MCMC parameter trace for the fit of the VFT equation to the normal vector ACF for derivative **V**.

S8 Corner plots

Note that all the traces presented in this section have been sub-sampled by the maximum autocorrelation time over all chains. Thus, the corner plots only contain uncorrelated values.

S8.1 Corner plots: Fitting the diffusivity

In the interest of space, we only show a representative corner plot for derivative **I** at 450 K (Fig. S16). All corner plots for the remianing perylene derivatives and temperatures are similar.

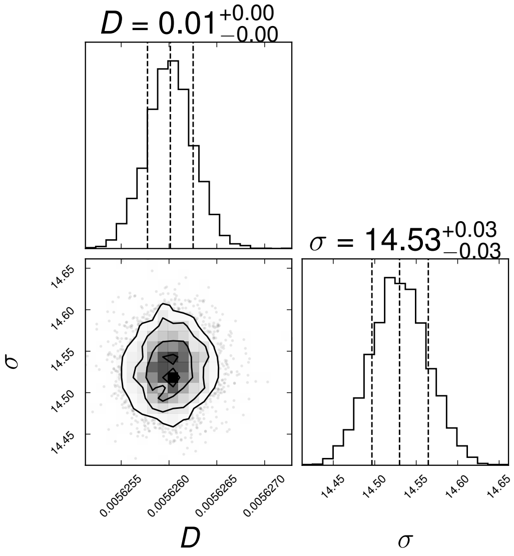

Figure S16: Corner plot of the MCMC parameter samples for fitting the diffusivity in equation (S5) to the MSD at 450 K for derivative **I**.

S8.2 Corner plots: Fitting the VFT equation to the diffusivity

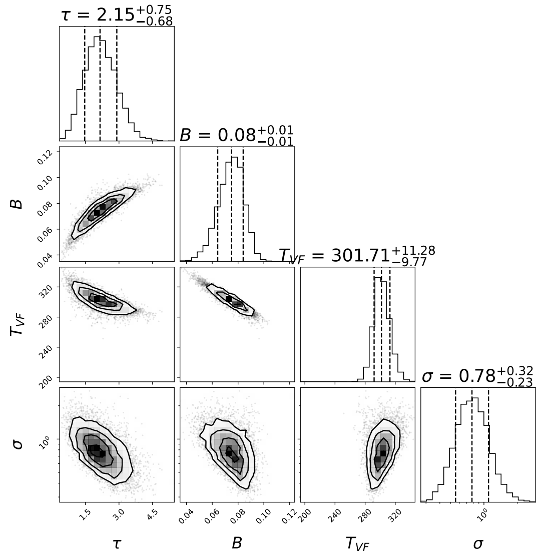

(a) Derivative **I**

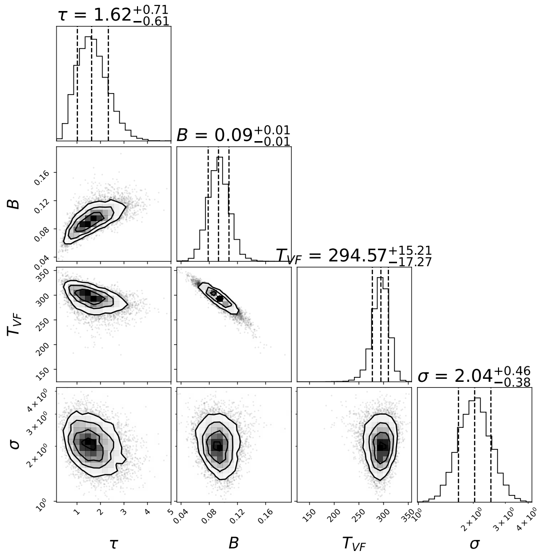

(b) Derivative **II**

Figure S17: Corner plot for the fit of the VFT equation to the diffusivity.

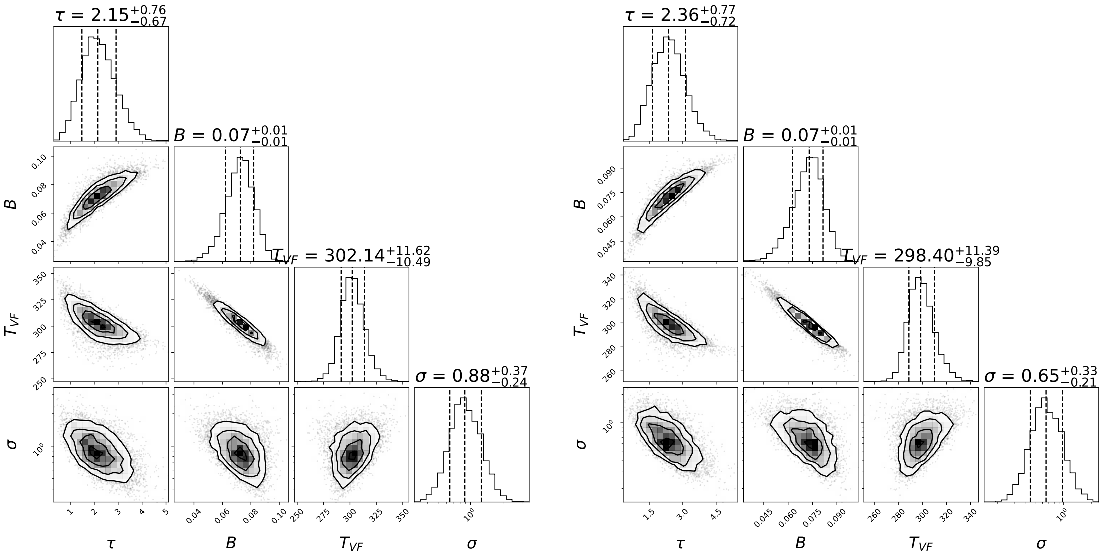

(a) Derivative **III** (b) Derivative **IV**

Figure S18: Corner plot for the fit of the VFT equation to the diffusivity.

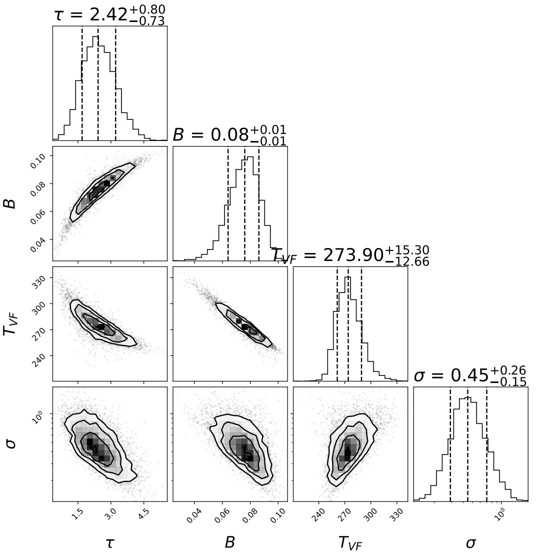

Figure S19: Corner plot for the fit of the VFT equation to the diffusivity for derivative **V**

S8.3 Corner plots: Fitting the normal vector ACF

In the interest of space, we only show a representative corner plot for derivative **I** at 450 K (Fig. S20). All corner plots for the remianing perylene derivatives and temperatures are similar.

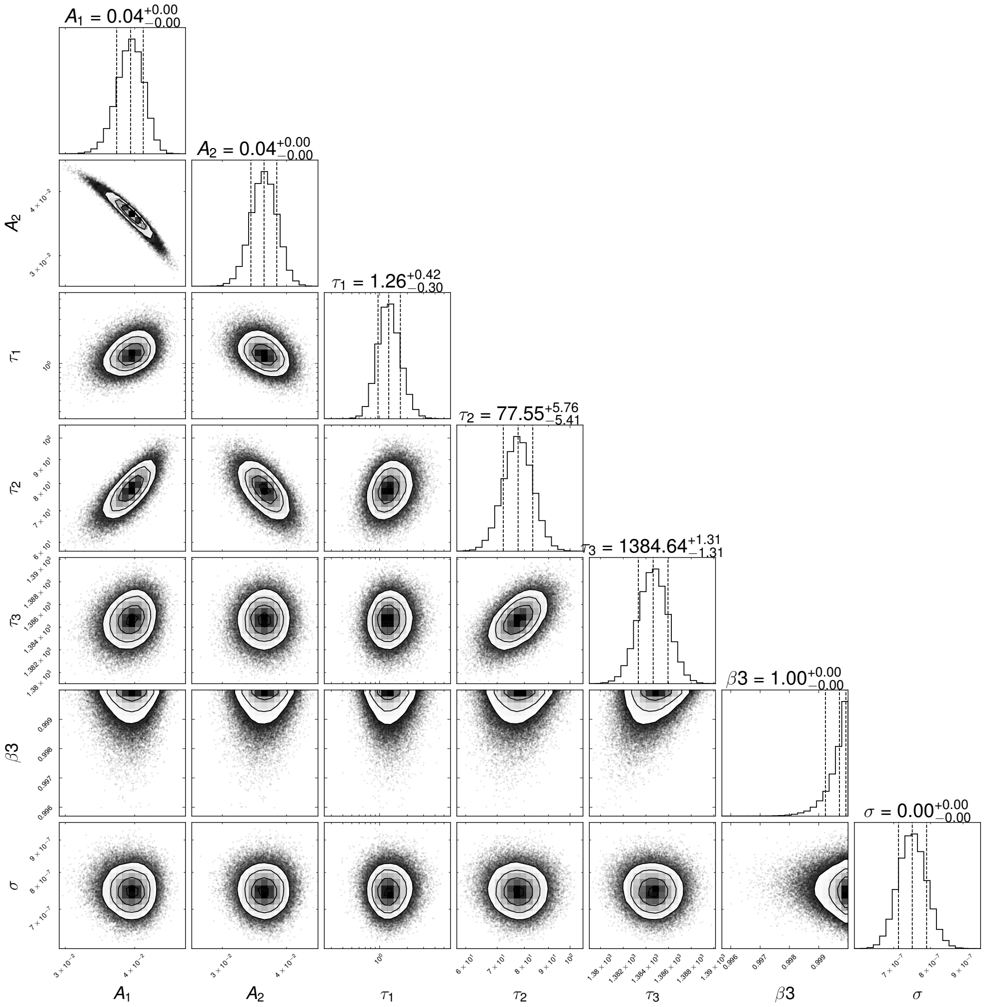

Figure S20: Corner plot of the MCMC parameter samples for fitting the triple exponential function in (S15) to the normal vector ACF at 450 K for derivative **I**.

S8.4 Corner plots: Fitting the VFT equation to the normal vector ACF

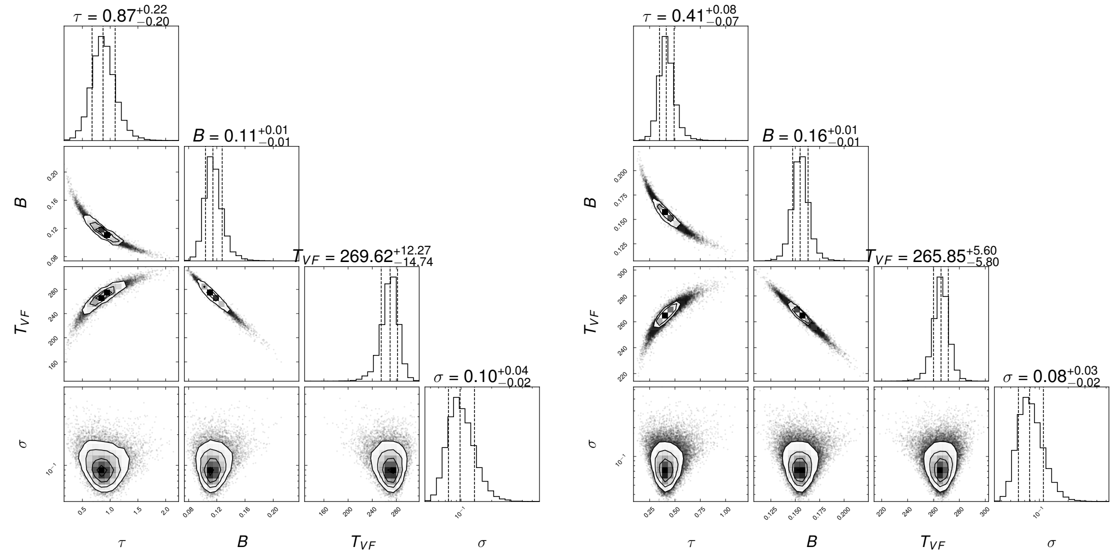

(a) Derivative **I** (b) Derivative **II**

Figure S21: Corner plot for the fit of the VFT equation to the normal vector ACF.

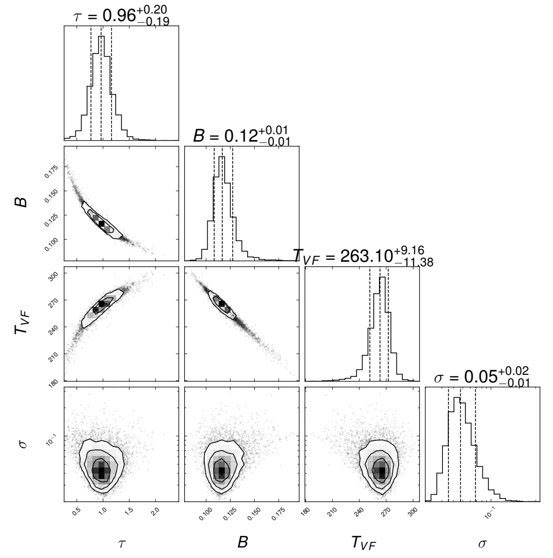

(a) Derivative **III**

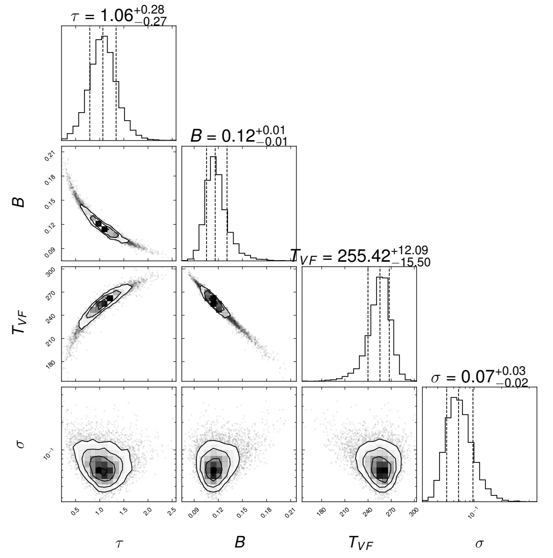

(b) Derivative **IV**

Figure S22: Corner plot for the fit of the VFT equation to the normal vector ACF.

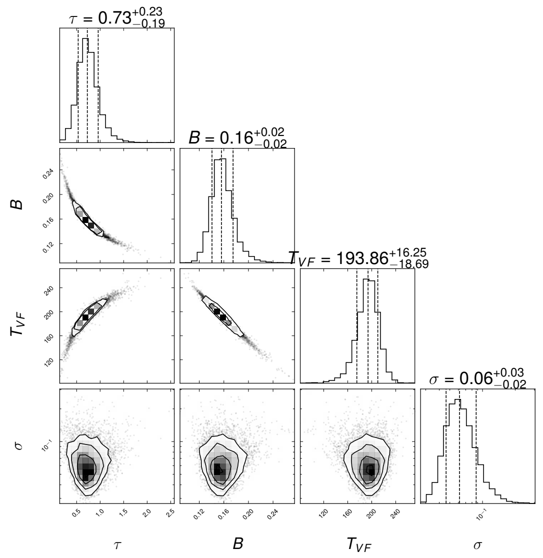

Figure S23: Corner plot for the fit of the VFT equation to the normal vector ACF for derivative **V**.

References

[1] Daan Frenkel and Berend Smit. *Understanding Molecular Simulation*. Academic Press, Inc., USA, 2nd edition, September 2001. ISBN 978-0-12-267351-1.

[2] Jacques Rault. Origin of the Vogel–Fulcher–Tammann law in glass-forming materials: The α–β bifurcation. *Journal of Non-Crystalline Solids*, 271(3):177–217, July 2000. ISSN 0022-3093. doi: 10.1016/S0022-3093(00)00099-5. URL `https://www.sciencedirect.com/science/article/pii/S0022309300000995`.

[3] Sandra Hultmark, Alex Cravcenco, Khushbu Kushwaha, Suman Mallick, Paul Erhart, Karl Börjesson, and Christian Müller. Vitrification of octonary perylene mixtures with ultralow fragility. *Science Advances*, 7(29):eabi4659, July 2021. ISSN 2375-2548. doi: 10.1126/sciadv.abi4659. URL `https://advances.sciencemag.org/lookup/doi/10.1126/sciadv.abi4659`.

[4] Nahid Farzi and Maede Ebrahim. Mechanical properties and glass transition temperature of metal-organic framework-filled epoxy resin: A molecular dynamics study. *Materials Chemistry and Physics*, 314:128874, February 2024. ISSN 0254-0584. doi: 10.1016/j.matchemphys.2023.128874. URL `https://www.sciencedirect.com/science/article/pii/S0254058423015821`.

[5] Navid Marchin, Shingo Urata, and Jincheng Du. Effect of three-body interaction on structural features of phosphate glasses from molecular dynamics simulations. *The Journal of Chemical Physics*, 161(15):154507, October 2024. ISSN 0021-9606. doi: 10.1063/5.0225188. URL `https://doi.org/10.1063/5.0225188`.

[6] Connor P. Callaway, Joel H. Bombile, Walker Mask, Sean M. Ryno, and Chad Risko. Thermomechanical enhancement of DPP-4T through purposeful π-conjugation disruption. *Journal of Polymer Science*, 60(3):559–568, 2022. ISSN 2642-4169. doi: 10.1002/pol.20210494. URL `https://onlinelibrary.wiley.com/doi/abs/10.1002/pol.20210494`.

[7] Kun-Han Lin, Leanne Paterson, Falk May, and Denis Andrienko. Glass transition temperature prediction of disordered molecular solids. *npj Computational Materials*, 7(1):1–7, November 2021. ISSN 2057-3960. doi: 10.1038/s41524-021-00647-w. URL `https://www.nature.com/articles/s41524-021-00647-w`.

[8] Paul N. Patrone, Andrew Dienstfrey, Andrea R. Browning, Samuel Tucker, and Stephen Christensen. Uncertainty quantification in molecular dynamics studies of the glass transition temperature. *Polymer*, 87:246–259, March 2016. ISSN 0032-3861. doi: 10.1016/j.polymer.2016.01.074. URL `https://www.sciencedirect.com/science/article/pii/S003238611630074X`.

[9] Samuel E. Root, Suchol Savagatrup, Christopher J. Pais, Gaurav Arya, and Darren J. Lipomi. Predicting the Mechanical Properties of Organic Semiconductors Using Coarse-Grained Molecular Dynamics Simulations. *Macromolecules*, 49(7):2886–2894, April 2016. ISSN 0024-9297. doi: 10.1021/acs.macromol.6b00204. URL `https://doi.org/10.1021/acs.macromol.6b00204`.
