# Supplementary figures and images for: Probing Glass Formation in Perylene Derivatives via Atomic-Scale Simulations and Bayesian Regression
Source: J Phys Chem B. 2025 Jun 23;129(26):6613–9. doi: 10.1021/acs.jpcb.5c00837 (PMC12235637; doi:10.1021/acs.jpcb.5c00837)

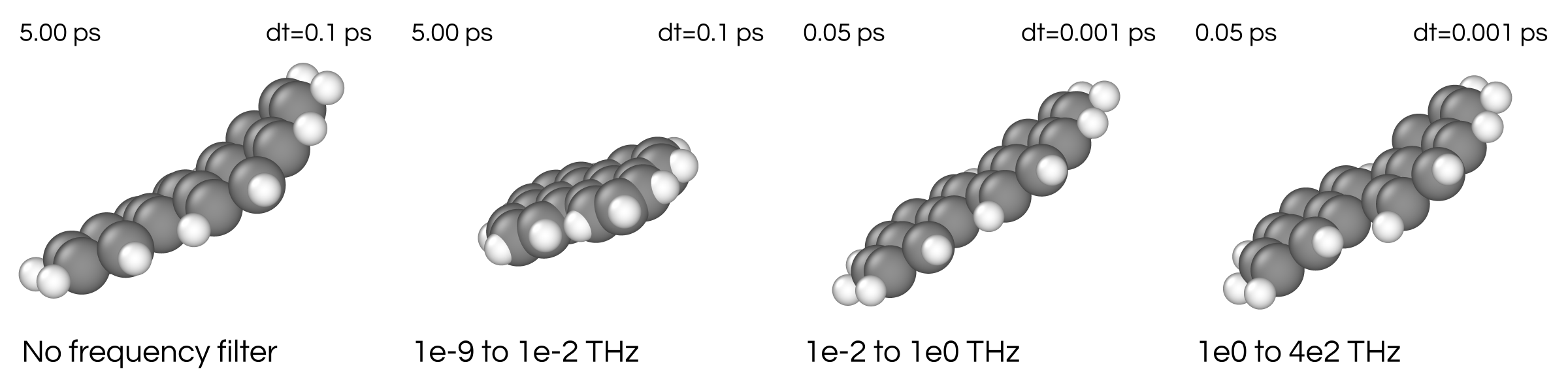

Supplement: Supplementary file 2 [file jp5c00837_si_002.gif]
